# The Respiratory Rate, Age, and Mean Arterial Pressure (RAM) Index: A Novel Prognostic Tool to Predict Mortality among Adult Patients with Acute Heart Failure in the Emergency Department

**DOI:** 10.3390/medicina60091423

**Published:** 2024-08-30

**Authors:** Yu Chang, Chan-Huan Peng, Jiann-Hwa Chen, Yu-Ting Lee, Meng-Yu Wu, Jui-Yuan Chung

**Affiliations:** 1 Department of Emergency Medicine, Cathay General Hospital, Taipei 106438, Taiwan; s0001049@gmail.com (Y.C.); chanhuan.peng@gmail.com (C.-H.P.); chenjiannhwa@yahoo.com.tw (J.-H.C.); j79312@hotmail.com (Y.-T.L.); 2 School of Medicine, Fu Jen Catholic University, Taipei 221037, Taiwan; 3 Department of Emergency Medicine, Taipei Tzu Chi Hospital, Buddhist Tzu Chi Medical Foundation, New Taipei City 231016, Taiwan; 4 School of Medicine, Tzu Chi University, Hualien 970374, Taiwan; 5 School of Medicine, National Tsing Hua University, Hsinchu 300044, Taiwan

**Keywords:** acute heart failure, emergency department, mortality, prognostic tool, physiological parameters

## Abstract

*Background and Objectives*: Acute heart failure (AHF) is a life-threatening condition frequently encountered in the emergency department (ED). Identifying reliable prognostic indicators for in-hospital mortality is crucial for risk stratification and the appropriate management of AHF patients. This study aimed to assess the most effective method for predicting in-hospital mortality among various physiological parameters in patients with AHF presenting to the ED. Additionally, the study evaluated the effectiveness of the RAM index—respiratory rate (RR), age, and mean arterial pressure (MAP)—derived from the shock index (SI) by replacing heart rate with RR, as a novel prognostic tool. This was compared with the SI and its other derivatives to predict in-hospital mortality in adult patients with AHF presenting to the ED. *Materials and Methods*: This is a retrospective study conducted in the ED of an urban medical center, enrolling adult patients with signs and symptoms of AHF, who met the epidemiological diagnosis criteria, between January 2017 and December 2021. Baseline physiological parameters, including the RR, heart rate, systolic blood pressure, and diastolic blood pressure, were recorded upon ED admission. The RAM index was calculated as the RR multiplied by the age divided by the MAP. Statistical analysis was performed, including univariate analysis, logistic regression, and receiver operating characteristic (ROC) curve analysis. *Results*: A total of 2333 patients were included in the study. A RAM index > 18.6 (area under ROC curve (AUROC): 0.81; 95% confidence interval (CI): 0.79–0.83) had a superior mortality discrimination ability compared to an SI > 0.77 (AUROC: 0.75; 95% CI: 0.72–0.77), modified shock index > 1.11 (AUROC: 0.75; 95% CI: 0.73–0.77), age shock index > 62.7 (AUROC: 0.74; 95% CI: 0.72–0.76), and age-modified shock index > 79.9 (AUROC: 0.75; 95% CI: 0.73–0.77). A RAM index > 18.6 demonstrated a 7.36-fold higher risk of in-hospital mortality with a sensitivity of 0.80, specificity of 0.68, and negative predictive value of 0.97. *Conclusions*: The RAM index is an effective tool to predict mortality in AHF patients presenting to the ED. Its superior performance compared to traditional SI-based parameters suggests that the RAM index can aid in risk stratification and the early identification of high-risk patients, facilitating timely and aggressive treatment strategies.

## 1. Introduction

Heart failure (HF) is a global health concern affecting approximately 26 million individuals worldwide [1], leading to substantial hospital admissions annually across various countries, with numbers exceeding 40,000 in Taiwan, 80,000 in the United Kingdom, 200,000 in Japan, and 1 million in both the United States (U.S.) and Europe [2,3,4,5]. In Taiwan, the annual incidences of HF hospitalization were reported to be 2181 per 100,000 population in the elderly population (≥65 years) and 88 per 100,000 population in younger individuals (20–64 years) [6].

While the survival rates of HF patients have improved due to modern therapies [7,8,9,10], long-term prognosis remains unfavorable, with a significant proportion succumbing to the condition within one to five years [11]. Moreover, the readmission rate within the first three months after hospitalization for acute heart failure (AHF) is about 30% in the U.S. and other countries [4,5]. In contrast, mortality rates during hospitalization for HF range from 2% to 17%, underscoring the severity of the condition [11].

A primary diagnosis of AHF accounts for approximately one million emergency department (ED) visits in the U.S. with notable morbidity and mortality implications [12]. Extensive research has focused on the clinical, biochemical, and echocardiographic indexes that influence the prognosis of AHF [13] as well as identified several risk prediction scores for predicting mortality and other adverse events in ED AHF patients [14]. However, there has been a relative lack of emphasis on the utilization of physiological parameters, which offer the advantages of being easily obtained and cost effective.

The shock index (SI) and its derivatives, such as modified SI (MSI), age SI (ASI), and age-modified SI (AMSI), have demonstrated reliable predictive capabilities for in-hospital mortality in AHF patients [15]. Additionally, novel derivative indices, such as the heart rate (HR)-to-respiratory rate (RR) ratio and respiratory efficacy index (REFI = RR × 100/SatO_2_), have shown promise in predicting mortality and intensive care unit (ICU) admissions [16].

The primary objective of this study is to determine the most effective method for predicting in-hospital mortality among various physiological parameters in patients with AHF presenting to the ED. Additionally, the study aims to evaluate the prognostic value of the newly proposed RAM index—RR, age, and mean arterial pressure (MAP)—compared to the traditional SI and its derivatives in predicting in-hospital mortality among adult patients with AHF presenting to the ED.

## 2. Methods

### 2.1. Study Population

This is a retrospective study conducted in Cathay General Hospital, an urban medical center in Taipei City, Taiwan. The hospital has a capacity of 800 beds and an estimated annual ED visiting volume of 60,000. The study included adult patients who visited the ED and were subsequently hospitalized between January 2017 and December 2021, with signs and symptoms indicative of AHF.

**Figure 1:**
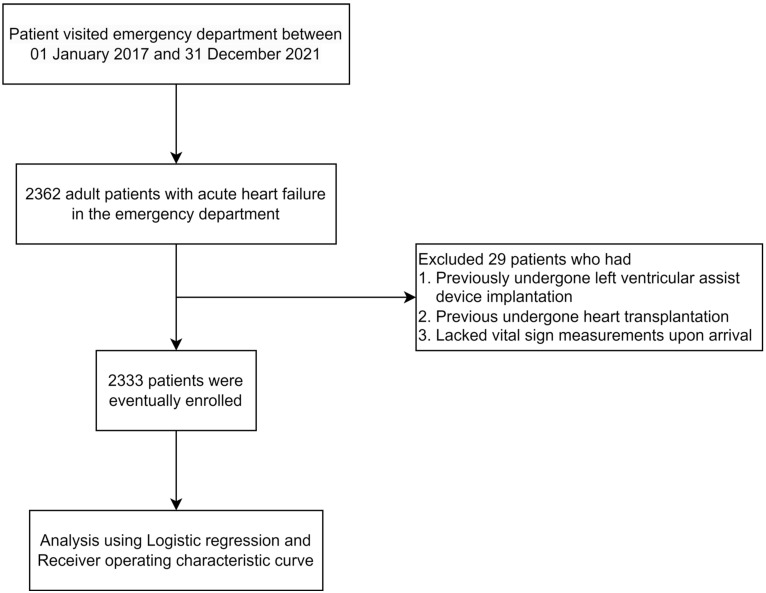
Flowchart of this study.

The inclusion criteria were as follows: adult patients aged 18 years or older who presented with signs and symptoms of AHF, including rapid deterioration of dyspnea or peripheral edema, cardiogenic shock, and lung congestion observed on chest X-rays at the time of their ED visit, who met the epidemiological diagnosis criteria, and were also diagnosed with AHF upon discharge (The International Statistical Classification of Diseases and Related Health Problems 10th Revision, ICD-9 codes 398.91, 402.01, 402.11, 402.91, 404.01, 404.03, 404.11, 404.13, 404.91, 404.93, 428.0, 428.01, 428.09, 786.50, 786.51, 786.52, 786.59, 786.09, and 786.05). AHF may be the first manifestation of HF (new onset) or, more frequently, be due to an acute decompensation of chronic HF. Patients who had previously undergone left ventricular assist device implantation or heart transplantation were excluded from the study. Furthermore, patients who lacked vital signs measurements upon arrival were also excluded from the study cohort (Figure 1).

### 2.2. Data Collection

Patient data between January 2017 and December 2021 were retrieved from the electronic medical record system. Data collection was performed from January 2023 to March 2023. The collected data encompassed various aspects, including patient demographics, comorbidities, vital signs, peripheral oxygen saturation (SpO_2_), Glasgow Coma Scale (GCS) score, echocardiography results, laboratory data, and length of hospitalization. The specific data items included the patient’s age (in years), gender (male/female), systolic and diastolic blood pressures (SBP and DBP, measured in mmHg), MAP (measured in mmHg), HR (measured in beats per minute), RR (measured in breaths per minute), SpO_2_ level (expressed as a percentage), GCS score, left ventricular ejection fraction (LVEF, represented as a percentage), and history of chronic diseases such as heart disease, diabetes mellitus, asthma or chronic obstructive pulmonary disease, cancer, and chronic kidney disease. The patients underwent an evaluation during triage in the emergency department, where a complete set of vital signs, including the SBP, DBP, HR, RR, and SpO_2_, were measured using a physiologic monitor. The MAP was calculated using the formula 1/3 × SBP + 2/3 × DBP, and the HR was measured for at least 20 s. Additionally, laboratory data consisting of the white blood cell (WBC) count (measured in 10^3^ cells/mm^3^), hemoglobin level (measured in g/dL), platelet count (measured in 10^3^/mm^3^), sodium level (measured in mEq/L), potassium level (measured in mEq/L), blood urea nitrogen (BUN) level (measured in mg/dL), creatinine level (measured in mg/dL), alanine aminotransferase (ALT) level (measured in IU/L), troponin I level (measured in ng/mL), and high-sensitivity troponin T (hs-Troponin T) level (measured in ng/L) were gathered. The duration of stay in the regular ward and intensive care unit (ICU) was also recorded. Physiological parameters, including the SBP, DBP, HR, RR, SpO_2_, and GCS score, were measured upon triage in the ED. Patients with missing data were excluded.

To calculate the shock indices for each patient, the following formulas were employed: the SI was determined by dividing the HR by the SBP; the MSI was obtained by dividing the heart rate by the MAP; the ASI was calculated by multiplying the age by the SI; and the AMSI was determined by multiplying the age by the MSI. A newly designed index, the RAM index, was developed via the basis of the AMSI formula with the replacement of HR with RR. The RAM index was calculated as the product of the RR and age divided by the MAP. The formula for the RAM index is as follows: RAM index = (RR × age)/MAP.

### 2.3. Endpoints

The primary outcome of this study was defined as the occurrence of in-hospital mortality following the presentation of patients to the ED.

### 2.4. Statistical Analysis

G-Power 3.0 was used to determine the power of the study with a sample size of 2333 patients, yielding a power of 0.80. Other statistical analyses were performed using MedCalc for Windows, version 20.218 (MedCalc Software, Ostend, Belgium). Baseline characteristics of the study participants were compared by dividing them into two groups: the survival group and the mortality group. To determine the distribution of continuous data, we used statistical tests such as the Shapiro–Wilk test and visual inspections of histograms and Q–Q plots. Normally distributed continuous data were presented as means ± standard deviations (SDs), while non-normally distributed continuous data were summarized as medians with the interquartile range (IQR). Univariate analysis for continuous variables was performed using independent samples t-test or the Mann–Whitney–Wilcoxon test. Categorical variables were analyzed using Pearson’s chi-squared test or Fisher’s exact test.

Logistic regression analysis was used to evaluate the mortality prediction abilities of the RAM index, SI, MSI, ASI, and AMSI in adult AHF patients. The optimal cutoff points for the RAM index, SI, MSI, ASI, and AMSI for predicting mortality in adult AHF patients were determined using the Youden index. The discriminatory abilities of the RAM index, SI, MSI, ASI, and AMSI for mortality prediction were assessed using the area under the receiver operating characteristic curve (AUROC). Multivariate logistic regression analysis with the forward selection procedure was conducted to adjust for potential confounders that could affect mortality (cancer and asthma or chronic obstructive pulmonary disease (COPD)). The reliability of the scoring systems was evaluated using the Hosmer–Lemeshow goodness-of-fit test. Additionally, the sensitivity, specificity, positive predictive value, and negative predictive value of the RAM index for predicting mortality in adult AHF patients were calculated.

### 2.5. Ethics

This study was approved by the Institutional Review Board of the Cathay General Hospital (No. CGH-P111048) and was conducted according to the tenets of the Declaration of Helsinki.

## 3. Results

A total of 2333 patients were included in this study, with a mean age of 76.53 ± 14.27 years and 54% being male. The overall in-hospital mortality rate was 10.3%. Table 1 provides a detailed overview of the baseline characteristics of all patients and each group. Variables such as the Glasgow Coma Scale (GCS) score and duration of ward and intensive care unit (ICU) stay were not normally distributed and are presented as medians with the interquartile range (IQR).

The most common comorbidities observed in the study population were heart disease (83.7%), hypertension (69.3%), and chronic kidney disease (63.9%), while the percentages of asthma or chronic obstructive pulmonary disease (COPD) and cancer were significantly higher in the mortality group compared to the survival group (*p* < 0.05).

The mean ± SD values for the SBP, DBP, MAP, HR, and RR were 143.87 ± 32.47 mmHg, 79.07 ± 19.9 mmHg, 100.33 ± 21.85 mmHg, 93.38 ± 24.4 beats per minute, and 21.77 ± 4.24 breaths per minute, respectively. The mortality group exhibited lower SBP (133.60 ± 31.95 mmHg), DBP (73.10 ± 19.87 mmHg), and MAP (92.90 ± 21.68 mmHg) values compared to the survival group (145.06 ± 32.32 mmHg, 79.76 ± 19.80 mmHg, and 101.19 ± 21.71 mmHg, respectively). The mortality group also had a significantly higher RR (26.58 ± 4.42 breaths per minute) compared to the survival group (21.22 ± 3.85 breaths per minute; *p* < 0.01). Although not statistically significant (*p* = 0.07), the mortality group had a slightly higher heart rate (96.37 ± 27.45 beats per minute) compared to the survival group (93.04 ± 24.01 beats per minute). Additionally, the GCS score was lower in the mortality group (15 [the interquartile range: 11–15]) compared to the survival group (15 [the interquartile range: 15–15]); *p* < 0.01). Moreover, the shock indices (SI, MSI, ASI, and AMSI) were higher in the mortality group (*p* < 0.01). The RAM index was 24.58 ± 7.16 in the mortality group and 16.69 ± 5.83 in the survival group (*p* < 0.01). If calculated solely using the age divided by the MAP, the resulting values are higher in the mortality group compared to the survival group, with 0.93 ± 0.24 and 0.79 ± 0.24, respectively (*p* < 0.01).

Laboratory data analysis revealed that the mortality group had higher WBC counts, serum potassium, BUN, and hs-Troponin T levels compared to the survival group (*p* < 0.01). The hemoglobin and serum sodium levels were both lower in the mortality group (*p* < 0.05). The ejection fraction measured using echocardiography did not differ significantly between the two groups (*p* = 0.49).

The comparison of mortality rate prediction among RAM, SI, MSI, ASI, AMSI, and age/MAP, as conducted using multivariate logistic regression analysis while adjusting for potential confounders (*p* < 0.10), including body temperature, GCS score, hypertension, diabetes mellitus, asthma or COPD, cancer, WBC, hemoglobin, sodium, potassium, BUN, and hs-Troponin T, revealed a higher odds ratio (OR) associated with the RAM index (3.47, *p* < 0.01) (Table 2).

**Figure 2:**
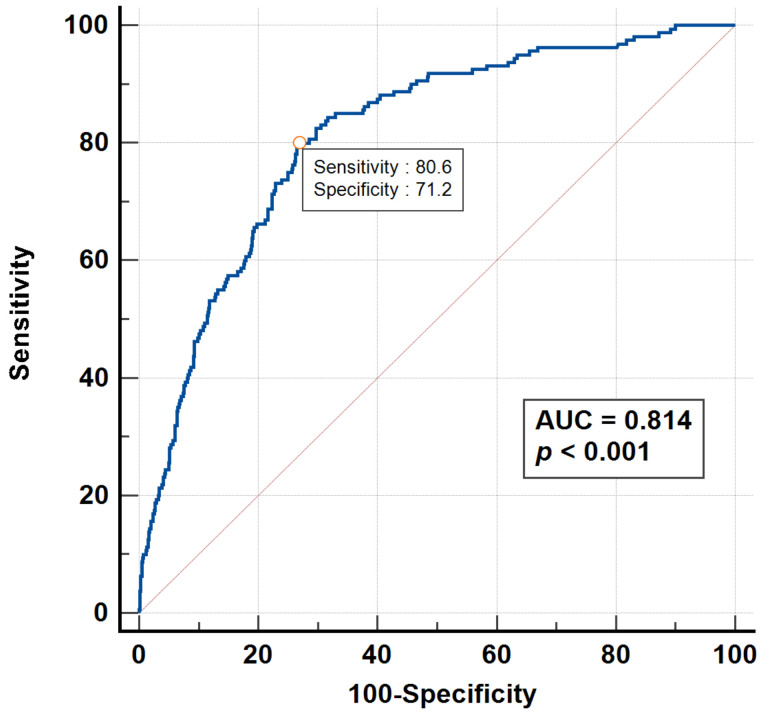
AUROC of RAM index > 18.6 to discriminate in-hospital mortality in adult ED patients with acute heart failure.

**Figure 3:**
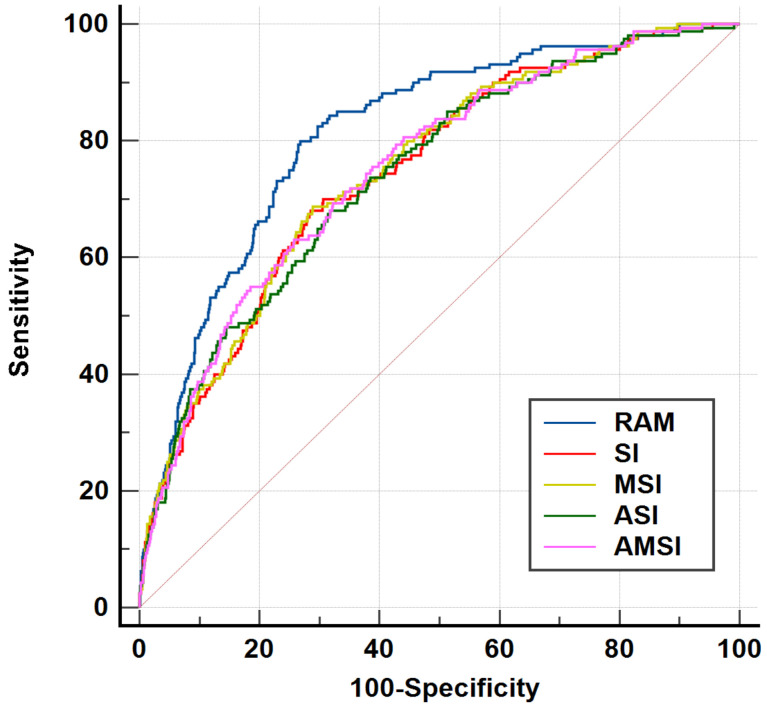
AUROCs of RAM, SI, MSI, ASI, and AMSI to predict mortality in adult ED patients with acute heart failure.

The optimal cutoff levels for the RAM index, SI, MSI, ASI, and AMSI in predicting in-hospital mortality in adult AHF patients were determined, using the Youden index, to be 18.6, 0.77, 1.11, 62.7, and 79.9, respectively. Logistic regression analysis revealed that adult AHF patients with a RAM index > 18.6 had an 8.15-fold higher risk of mortality (Table 3). The bootstrapped OR for predicting mortality was analyzed and presented in Supplementary Table S1. The bootstrapped OR for the RAM score’s predictive capability was determined to be 8.15. The adjusted area under the receiver operating characteristic (AUROC) analysis, accounting for potential confounders (*p* < 0.10), indicated that a RAM index > 18.6 (AUROC: 0.81, 95% CI: 0.79–0.83, Figure 2) had a superior mortality discrimination ability compared to an SI > 0.77 (AUROC: 0.75; 95% CI: 0.72–0.77), MSI > 1.11 (AUROC: 0.75; 95% CI: 0.73–0.77), ASI > 62.7 (AUROC: 0.74; 95% CI: 0.72–0.76), and AMSI > 79.9 (AUROC: 0.75; 95% CI: 0.73–0.77) (Table 4 and Figure 3).

Among young patients (<65 years old, 447 patients), there were 23 mortalities. In these 23 patients, 8 patients met the criteria for a high RAM index (>18.6). The sensitivity was 0.35, with a 95% CI from 0.16 to 0.57. The specificity was 0.98, with a 95% CI from 0.96 to 0.99.

The performance of the RAM index > 18.6 in predicting in-hospital mortality in adult AHF patients demonstrated a sensitivity of 0.80 (95% CI: 0.75–0.85), specificity of 0.68 (95% CI: 0.65–0.70), positive predictive value of 0.22 (95% CI: 0.21–0.24), and negative predictive value of 0.97 (95% CI: 0.96–0.97) (Table 5).

## 4. Discussion

In the present study, the RAM index demonstrated superior predictive performance for in-hospital mortality in AHF patients compared to the SI, MSI, ASI, and ASMI, with an adjusted AUROC of 0.81. Unlike traditional shock index-based parameters, the RAM index incorporates the RR into the formula, recognizing that high RRs are linked to poor outcomes in AHF patients. This is supported by a study showing that the RR, when combined with other indices, provides a high predictive accuracy for mortality and ICU admissions in AHF patients [16]. Furthermore, in the clinical setting, physicians could interpret a RAM score greater than 18.6 in AHF patients as indicating an 8.15-fold increased risk of mortality, while those with a RAM score lower than 18.6 would have a 97% survival rate.

The concept of the SI was first introduced in 1967 as a tool for managing hemorrhagic shock [17]. It is calculated by dividing the heart rate by the systolic blood pressure and serves as a quick and noninvasive predictor of mortality in patients admitted to the ED. Experimental and clinical studies have demonstrated a linear inverse relationship between the SI and physiological parameters such as the cardiac index, stroke volume, left ventricular stroke work, and MAP [18]. In 1994, Rady et al. found that an SI ≥ 0.9 was associated with higher illness severity, increased hospital admission rates, and a higher likelihood of intensive therapy upon admission compared to pulse or blood pressure alone [19]. However, older patients may have higher baseline systolic blood pressure even after injury, which could underestimate the severity of underlying shock in older traumatized patients [20]. This consideration led to the idea of enhancing the original SI by incorporating additional parameters. Zarzaur et al. proposed multiplying the SI by the age as one solution [21]. Replacing blood pressure with the MAP, as adopted in our study, transforms the SI into the MSI, with prior research suggesting that the MSI offers improved mortality prediction over the SI and other vital signs in emergency patients [22]. Other shock index-based parameters, including the SI, ASI, modified SI, and AMSI, are supported by Bondariyan et al. as simple bedside tools with reliable predictive capabilities for in-hospital mortality [15].

Although shock index-based parameters have shown strong prognostic capabilities, some components may lead to misleading results in AHF patients. Since heart rate is an essential component of the SI, factors like increasing age, biological changes such as fibrosis of the sinus atrial node, decreased adrenergic sensitivity, and reduced responsiveness to autonomic cardiovascular reflexes may result in a lower heart rate, particularly in older patients [23]. This could lead to underestimating the severity of their condition, and several studies have reported inconsistent outcomes as a result [24,25].

Beyond shock index-based parameters, Adrien Basset et al. developed the Brest score, a clinical prediction tool specifically designed for diagnosing congestive heart failure (CHF) in the emergency care setting. This score uses symptoms such as sudden dyspnea, night episodes, and orthopnea to estimate the likelihood of CHF in adult patients presenting with dyspnea, distinguishing it from other potential causes [26]. Recently, new approaches have emerged for understanding the shortness of breath experienced by AHF patients requiring hospitalization. For predicting 90-day mortality, plasma BUN and hemoglobin levels were found to be predictive primarily in younger patients, while the RR and albumin levels were more strongly associated with mortality in older patients [27]. In our study, the RAM index was developed based on the SI formula, replacing HR—which did not show a significant difference between the mortality and survival groups (*p* = 0.07)—with RR, as the RR was significantly higher in the mortality group compared to the survival group. By incorporating the RR, the RAM index offers a more comprehensive assessment of risk and demonstrates better predictive performance for mortality outcomes. Similar findings have been observed in other studies. However, it is important to note that this association does not imply a direct causal effect on mortality rates.

The findings of the study highlighted the significance of the RR among various physiological parameters in adult patients presenting to the ED with AHF. Furthermore, our results suggest that the RAM index, which incorporates the RR, could be a valuable tool for stratifying patients based on their prognosis. By considering the RAM index, healthcare professionals can gain additional insight into the severity and potential outcomes of AHF patients, aiding in more effective risk stratification and treatment decision making in the ED setting. Based on our findings, we propose that patients with a RAM index above 18.6 should receive prompt and aggressive treatment to reduce in-hospital mortality. This treatment approach should involve identifying and addressing the underlying causes of AHF (such as acute coronary syndrome, hypertensive emergency, acute pulmonary embolism, or arrhythmias) that require urgent management. Additionally, criteria for ICU hospitalization should be evaluated, and early AHF management should include interventions such as oxygen therapy, diuretics, and vasodilators (in the absence of symptomatic hypotension) following the European Society of Cardiology (ESC) and American Heart Association (AHA) guidelines. Other medications like digoxin, amiodarone, and thromboembolism prophylaxis may also be considered according to the relevant guidelines. This comprehensive and aggressive treatment approach aims to optimize patient outcomes and mitigate the risk of in-hospital mortality. This might also explain why the SI did not exhibit a strong performance in the ROC curve analysis and was outperformed by the ASI, MSI, and particularly the RAM index.

In the present study, the ages and troponin-T levels were significantly higher in the mortality group compared to the survival group. This finding aligns with a retrospective study conducted by Peacock et al., which included a population of 84,872 hospitalized AHF patients. Their study demonstrated that patients with elevated troponin-T levels at admission had higher in-hospital mortality compared to those with non-elevated troponin-T levels. In contrast, the percentage of LVEF in the current study did not show a significant difference between the mortality and survival groups. This is consistent with several studies that have questioned the prognostic value of LVEF in AHF patients. For instance, Gómez-Otero et al. investigated the baseline characteristics and outcomes of patients admitted with different LVEF levels (LVEF 40–49%, LVEF < 40%, and LVEF ≥ 50%). They found that patients with LVEF 40–49% shared characteristics with both the LVEF < 40% and LVEF ≥ 50% groups. While the clinical characteristics of the LVEF 40–49% group were closer to those of the LVEF ≥ 50% group, after one year of follow-up, there were no significant differences in total mortality, causes of death, or hospital readmissions for HF among the LVEF groups [28]. Furthermore, acutely measured LVEF may be labile and may not accurately reflect a patient’s chronic cardiac status. Additionally, the correlation of LVEF with hemodynamic, clinical, and neurohormonal measures is limited in patients with AHF [29].

Several limitations should be acknowledged in the study. Firstly, it was a single-center study, which may limit the generalizability of our findings to the broader population of patients with AHF. However, the characteristics of our study population were similar to those observed in large registries, which provide some reassurance regarding the representativeness of our sample. Nevertheless, caution is advised when extrapolating the results to other settings, and external validation is necessary. Secondly, the retrospective nature of our analysis introduces inherent limitations. Although we employed systematic recruitment using computerized medical records, retrospective studies are susceptible to biases and confounding variables that may impact the validity of the findings; these include the urine output, fluid balance, skin color/temperature/turgor, edema, and detailed treatment and intervention information. Prospective studies with careful data collection and standardized protocols would provide more robust evidence. Additionally, due to health insurance policies in Taiwan, some laboratory data, including blood lactate, pro-BNP, and arterial gas analysis, which may play an important role in prognosis indicators, were not fully obtained for a large group of our patients. Thirdly, our assessment of physiological parameters was based on a single measurement taken at the time of presentation to the triage team in the ED. This approach limited our ability to analyze changes in these parameters over time or assess their dynamic nature during the hospital course. Multiple measurements at different time points would have provided a more comprehensive understanding of the physiological status of the patients.

Moreover, it is important to acknowledge that specific information regarding the medications used by the participants was not collected, potentially introducing confounding effects on the physiological parameters and extended laboratory data about organ function changes in kidney (including estimated glomerular filtration rate), liver, and heart biomarkers. Additionally, baseline heart rhythm data of the patients were not included and analyzed in our study. Consequently, the potential impact of these medications and heart rhythm on the study outcomes remains unaccounted for in our analysis. While treatment decisions were guided by our cardiovascular experts, it is noteworthy that the medical treatment and dosage administered during hospitalization in the studied group were not accounted for, which could potentially play a fundamental role in patient outcomes.

Although SARS-CoV-2 was first confirmed to have spread to Taiwan on 21 January 2020, only 823 confirmed COVID-19 cases, including nine deaths, were reported to the Taiwan CDC in 2020. In 2021, there were a total of 16,303 confirmed cases, including 842 deaths. In comparison, the larger outbreak began in 2022, resulting in a total of 9,167,811 cases, including 15,755 deaths [30]. Therefore, although the study period includes data from 2020 to 2021, the data were minimally influenced by the COVID-19 pandemic.

Finally, unmeasured variables such as the duration of AHF, socioeconomic factors, obesity, and lactate level were not included in our analysis, and their potential impact on the study results cannot be ruled out. Future studies could consider including these variables to provide a more comprehensive assessment of the factors influencing outcomes in patients with AHF.

## 5. Conclusions

In conclusion, our study highlights the value of the RAM index, incorporating the RR, as an effective prognostic tool for in-hospital mortality in adult patients presenting to the ED with AHF. The RAM index demonstrated superior predictive performance compared to the SI and its derivatives, suggesting its utility in risk stratification and the early identification of high-risk patients. While this association is notable, it is important to recognize that our study does not establish a causal relationship. Prompt aggressive treatment strategies should be considered for patients with a RAM index above 18.6 to reduce in-hospital mortality, including targeted management of underlying causes, ICU admission criterion evaluation, and adherence to evidence-based guidelines.

## Figures and Tables

**Table 1:** Characteristics of adult patients with acute heart failure in the emergency department.

Characteristics (n/%)	Total Patients (2333/100%)	Survived (2092/89.67%)	Died (241/10.33%)	*p*-Value
Age (years)	76.53 ± 14.27	75.88 ± 14.40	82.20 ± 11.70	<0.01
Sex (Male)	1259 (53.96)	1130 (54.01)	129 (53.53)	0.89
Triage	2 (2–3)	2 (2–3)	2 (1–2)	<0.01
Vital signs				
SBP (mmHg)	143.87 ± 32.47	145.06 ± 32.32	133.60 ± 31.95	<0.01
DBP (mmHg)	79.07 ± 19.9	79.76 ± 19.80	73.10 ± 19.87	<0.01
MAP (mmHg)	100.33 ± 21.85	101.19 ± 21.71	92.90 ± 21.68	<0.01
Heart rate (beats/min)	93.38 ± 24.4	93.04 ± 24.01	96.37 ± 27.45	0.07
Body temperature (°C)	36.72 ± 0.77	36.70 ± 0.75	36.83 ± 0.95	0.05
Respiratory rate (/min)	21.77 ± 4.24	21.22 ± 3.85	26.58 ± 4.42	<0.01
SpO_2_ (%)	93.76 ± 7.32	94.16 ± 6.70	90.36 ± 10.80	<0.01
GCS score	15 (15–15)	15 (15–15)	15 (11–15)	<0.01
Score				
RAM	17.50 ± 6.44	16.69 ± 5.83	24.58 ± 7.16	<0.01
SI	0.68 ± 0.24	0.67 ± 0.23	0.76 ± 0.30	<0.01
MSI	0.96 ± 0.31	0.95 ± 0.29	1.08 ± 0.39	<0.01
ASI	51.77 ± 20.31	50.59 ± 19.58	61.99 ± 23.49	<0.01
AMSI	73.63 ± 273.4	71.94 ± 26.25	88.35 ± 31.89	<0.01
Age/MAP	0.79 ± 0.24	0.79 ± 0.24	0.93 ± 0.24	<0.01
Past History				
Hypertension	1616 (69.27)	1480 (70.75)	136 (56.43)	<0.01
Diabetes mellitus	1049 (44.96)	955 (45.65)	94 (39.00)	0.05
Heart disease *	1953 (83.71)	1749 (83.60)	204 (84.65)	0.68
Asthma or COPD	582 (24.95)	500 (23.90)	82 (34.02)	<0.01
Chronic kidney disease	1493 (63.99)	1338 (63.96)	155 (64.32)	0.91
Cancer	491 (21.05)	416 (19.89)	75 (31.12)	<0.01
Laboratory data				
WBC (10^3^ cells/mm^3^)	9.54 ± 4.36	9.36 ± 4.12	11.06 ± 5.85	<0.01
Hemoglobin (g/dL)	11.40 ± 2.68	11.47 ± 2.68	10.80 ± 2.55	<0.01
Platelet (10^3^/mm^3^)	219.74 ± 91.35	219.24 ± 88.79	224.05 ± 111.18	0.52
Sodium (mmol/L)	134.74 ± 6.33	134.87 ± 5.94	133.64 ± 8.95	0.04
Potassium (mmol/L)	4.20 ± 0.77	4.17 ± 0.74	4.47 ± 0.95	<0.01
BUN (mg/dL)	41.27 ± 28.11	40.31 ± 27.54	49.54 ± 31.53	<0.01
Creatinine (mg/dL)	2.43 ± 2.44	2.44 ± 2.51	2.41 ± 1.79	0.80
ALT (IU/L)	39.56 ± 127.14	38.46 ± 129.36	49.15 ± 105.64	0.15
Troponin I (ng/mL)	0.48 ± 2.12	0.42 ± 1.83	0.92 ± 3.68	0.24
hs-Troponin T (ng/L)	144.84 ± 397.04	129.32 ± 344.85	285.16 ± 699.12	<0.01
Echocardiography				
EF	47.16 ± 18.74	47.07 ± 18.83	47.94 ± 17.92	0.49
Admission ^†^				
Ward days	9 (5–15)	9 (5–15)	8 (2–19)	0.01
ICU days	0 (0–2)	0 (0–2)	0 (0–10)	<0.01

Data are presented as %. Data with normal distribution are displayed as mean ± standard deviation; data that are not normally distributed are displayed as medians (interquartile range). SBP, systolic blood pressure; DBP, diastolic blood pressure; MAP, mean arterial pressure; SpO_2_, saturation of peripheral oxygen; GCS, Glasgow coma scale; SI, shock index; MSI, modified shock index; ASI, Age multiplied by shock index; AMSI, age multiplied by modified shock index; RAM, respiratory rate multiplied by age and divided by mean arterial pressure; WBC, white blood cell; BUN, blood urea nitrogen; ALT, alanine aminotransferase; hs-Troponin T, high-sensitivity Troponin T; EF, ejection fraction; ICU, intensive care unit. * Heart disease includes coronary artery disease and congestive heart failure. ^†^ Admission to general ward or intensive care unit.

**Table 2:** Mortality rate prediction comparison among RAM, SI, MSI, ASI, AMSI, and age/MAP using multivariate logistic regression analysis, accounting for potential confounders.

	Adjusted Odds Ratio	95% CI	*p*-Value	AUROC
RAM	3.47	2.89–4.16	<0.01	0.85
SI	2.26	1.94–2.62	<0.01	0.75
MSI	2.28	1.96–2.65	<0.01	0.75
ASI	2.29	1.97–2.66	<0.01	0.75
AMSI	2.32	1.99–2.70	<0.01	0.76
Age/MAP	2.19	1.89–2.54	<0.01	0.75

CI: confidence interval; AUROC, area under the curve; RAM, respiratory rate multiplied by age and divided by mean arterial pressure; SI, shock index; MSI, modified shock index; ASI, age multiplied by shock index; AMSI, age multiplied by modified shock index; MAP, mean arterial pressure.

**Table 3:** Mortality rate prediction comparison among RAM > 18.6, SI > 0.77, MSI > 1.11, ASI > 62.7, and AMSI > 79.9, performed using logistic regression analysis.

	Adjusted Odds Ratio	95% CI	*p*-Value
RAM > 18.6	8.15	5.88–11.30	<0.01
SI > 0.77	2.11	1.60–2.78	<0.01
MSI > 1.11	2.41	1.83–3.18	<0.01
ASI > 62.7	2.85	2.17–3.75	<0.01
AMSI > 79.9	2.88	2.20–3.77	<0.01

CI: confidence interval; RAM, respiratory rate multiplied by age and divided by mean arterial pressure; SI, shock index; MSI, modified shock index; ASI, age multiplied by shock index; AMSI, age multiplied by modified shock index.

**Table 4:** Covariate-adjusted receiver operating characteristic curve for mortality discrimination of RAM > 18.6, SI > 0.77, MSI > 1.11, ASI > 62.7, and AMSI > 79.9 in adult patients with acute heart failure in the emergency department.

	AUROC	95% CI	SE
RAM > 18.6	0.81	0.79–0.83	0.02
SI > 0.77	0.75	0.72–0.77	0.02
MSI > 1.11	0.75	0.73–0.77	0.02
ASI > 62.7	0.74	0.72–0.76	0.02
AMSI > 79.9	0.75	0.73–0.77	0.02

AUROC, area under the curve; CI, confidence interval; SE, standard error; RAM, respiratory rate multiplied by age and divided by mean arterial pressure; SI, shock index; MSI, modified shock index; ASI, age multiplied by shock index; AMSI, age multiplied by modified shock index.

**Table 5:** Performance of RAM > 18.6 in predicting mortality in adult patients with acute heart failure in the emergency department.

Performance	RAM > 18.6
Sensitivity	0.80 (0.74–0.85)
Specificity	0.68 (0.65–0.70)
Positive predictive value	0.22 (0.21–0.24)
Negative predictive value	0.97 (0.96–0.97)

RAM, respiratory rate multiplied by age and divided by mean arterial pressure.

## Data Availability

The datasets used and/or analyzed regarding the current study are available from the corresponding author upon reasonable request.

## Supplementary Materials

The following supporting information can be downloaded at: https://www.mdpi.com/article/10.3390/medicina60091423/s1, Table S1: Mortality rate prediction comparison between RAM > 18.6, SI > 0.77, MSI > 1.11, ASI > 62.7, and AMSI > 79.9, identified by multivariate logistic regression analysis (Bootstrapped odds ratio).
