# Supplementary material for: The Respiratory Rate, Age, and Mean Arterial Pressure (RAM) Index: A Novel Prognostic Tool to Predict Mortality among Adult Patients with Acute Heart Failure in the Emergency Department
Source: Medicina (Kaunas). 2024 Aug 30;60(9):1423. doi: 10.3390/medicina60091423 (PMC11433796; doi:10.3390/medicina60091423)
Supplement: Supplementary file 1 [file medicina-60-01423-s001.zip › Supplementary Table S1.pdf]

Supplementary Table S1: Mortality rate prediction comparison between RAM > 18.6, SI > 0.77, MSI > 1.11, ASI > 62.7, and AMSI > 79.9, identified by multivariate logistic regression analysis (Bootstrapped odds ratio).

	Bootstrapped OR	SE	Bootstrapped 95% CI*	p-Value
RAM > 18.6	8.15	1.45	5.93-11.6	<0.01
SI > 0.77	2.11	0.30	1.61-2.79	<0.01
MSI > 1.11	2.42	0.34	1.85-3.17	<0.01
ASI > 62.7	2.85	0.40	2.16-3.72	<0.01
AMSI > 79.9	2.88	0.40	2.20-3.75	<0.01

RAM, Respiratory rate multiplied by Age and divided by Mean arterial pressure; SI, Shock Index; MSI, Modified shock index; ASI, Age multiplied by shock Index; AMSI, Age multiplied by modified shock Index; OR, Odds ratio; SE, standard error; CI, confidence interval.

* Unless otherwise noted, bootstrap results are based on 2000 bootstrap samples
